# TGF-β2-Induced Invadosomes in Human Trabecular Meshwork Cells

**DOI:** 10.1371/journal.pone.0070595

**Published:** 2013-08-05

**Authors:** Hong Han, Daniel Kampik, Franz Grehn, Günther Schlunck

**Affiliations:** 1 Department of Ophthalmology, Würzburg University Hospital, Würzburg, Germany; 2 University College London, Institute of Ophthalmology, London, United Kingdom; 3 Department of Ophthalmology, Freiburg University Hospital, Freiburg, Germany; Northwestern University, United States of America

## Abstract

Primary open-angle glaucoma (POAG) is a leading cause of blindness due to chronic degeneration of retinal ganglion cells and their optic nerve axons. It is associated with disturbed regulation of intraocular pressure, elevated intraocular levels of TGF-β2, aberrant extracellular matrix (ECM) deposition and increased outflow resistance in the trabecular meshwork (TM). The mechanisms underlying these changes are not fully understood. Cell-matrix interactions have a decisive role in TM maintenance and it has been suggested that TGF-β-induced inhibition of matrix metalloproteases may drive aberrant ECM deposition in POAG. Invadopodia and podosomes (invadosomes) are distinct sites of cell-matrix interaction and localized matrix-metalloprotease (MMP) activity. Here, we report on the effects of TGF-β2 on invadosomes in human trabecular meshwork cells. Human TM (HTM) cells were derived from donor tissue and pretreated with vehicle or TGF-β2 (2 ng/ml) for 3d. Invadosomes were studied in ECM degradation assays, protein expression and MMP-2 activity were assessed by western blot and zymography and ECM protein transcription was detected by RT-qPCR. HTM cells spontaneously formed podosomes and invadopodia as detected by colocalization of Grb2 or Nck1 to sites of gelatinolysis. Pretreatment with TGF-β2 enhanced invadosomal proteolysis and zymographic MMP-2 activity as well as MMP-2, TIMP-2 and PAI-1 levels in HTM cell culture supernatants. Rho-kinase inhibition by H1152 blocked the effects of TGF-β2. Concomitant transcription of fibronectin and collagens-1, -4 and -6 was increased by TGF-β2 and fibrillar fibronectin deposits were observed in areas of invadosomal ECM remodelling. In contrast to a current hypothesis, our data indicate that TGF-β2 induces an active ECM remodelling process in TM cells, characterized by concurrent increases in localized ECM digestion and ECM expression, rather than a mere buildup of material due to a lack of degradation. Invadosomal cell adhesion and signaling may thus have a role in POAG pathophysiology.

## Introduction

The trabecular meshwork (TM) serves as a dynamic resistor to control the outflow of intraocular fluid and regulate intraocular pressure. Primary open-angle glaucoma (POAG) is associated with characteristic deposits of extracellular matrix in the TM and a subsequent increase in outflow resistance [1], [2]. The extent of ECM deposition correlates with the severity of optic nerve damage [3]. The cause of these changes and the subsequent increase in outflow resistance are not fully elucidated, but strong evidence points to transforming growth factor beta (TGF-β) as having a major role. Elevated intraocular levels of TGF-β2 have been detected in POAG patients [4] and overexpression of TGF-β in an anterior chamber perfusion model induces characteristic ECM alterations and increases outflow resistance [5]. Earlier work has suggested that TGF-β suppresses matrix metalloprotease (MMP) activity due to enhanced PAI-1 expression in human TM cells [6] and thus contributes to aberrant ECM deposition in POAG. Mounting evidence indicates that cell-matrix interactions in the TM have a crucial role in POAG pathophysiology. Actin modulating drugs such as Rho-kinase (ROCK) inhibitors have been shown to lower intraocular pressure [7] and are currently being tested in clinical trials to treat glaucoma patients.

Invadosomes are distinct cell-matrix interaction sites providing for cell adhesion and localized matrix degradation [8], [9], [10]. They form as small, short-lived, punctate, often ring-shaped structures referred to as podosomes, or as larger protrusions termed invadopodia, which appear to have an important role in tumor cell migration and metastasis. First observed in Src-transformed fibroblasts [11], invadosomes have been detected in osteoclasts, macrophages, vascular endothelial cells and cancer cells *in vitro* [12] and in vascular endothelial cells *ex vivo* [13]. Invadosomes have an actin core and Rho-GTPases were shown to be relevant in invadosome formation [14]. Based on colocalization studies focusing on MMPs and cytoskeletal components, podosome- or invadopodia-like structures (PILS) were observed in porcine trabecular meshwork cells [15]. However, typical localized gelatinolytic activity as a mandatory characteristic of invadopodia or podosomes has not been demonstrated in this cell type. It was our goal to clarify the nature of PILS in human TM cells and to assess the effect of TGF-β and ROCK inhibitors on invadosomes in this cell type.

Our data indicate that human TM cells spontaneously form podosomes and invadopodia as determined by Grb2 or Nck1 colocalization. TGF-β2 enhanced invadosome formation and ECM digestion as well as ECM protein transcription. Thus, TGF-β2 induces an active tissue remodelling process characterized by coordinated degradation and de novo expression of ECM.

## Materials and Methods

### Cell Culture

Human trabecular meshwork tissue was derived from donor cornea rings and cells were cultivated according to methods published earlier [16], [17] with slight modifications. The donors were 81 (male), 84 (male) and 58 (female) years of age. The tenets of the Declaration of Helsinki were followed in all procedures and the study was approved by the institutional ethics committee of the Faculty of Medicine, Würzburg University, Würzburg, Germany (approval #169/08). Written informed consent for scientific use of the material had been obtained at the time of cornea donorship approval. In brief, donor rings were transferred from the storage medium and kept in Dulbecco's Modified Eagle's Medium (DMEM, PAA Laboratories GmbH; Pasching, Austria) supplemented with 10% heat inactivated fetal calf serum (FCS, Biochrom, Berlin, Germany), 100 U/ml penicillin and 100 µg/ml streptomycin (both from PAA) for 24 h. Under microscopic guidance, anterior and posterior incisions were placed to isolate the trabecular meshwork, which was then removed using forceps and cut into smaller sections. The tissue sections were placed in 24 well plates, covered with a glass coverslip to avoid floating and incubated in growth medium (as above). Confluent cell layers were passaged by trypsinization. From the second passage, FCS concentration was lowered to 3%. Cells were characterized by assessing baseline a-B-crystallin expression and increased myocilin expression after 7 days of dexamethasone treatment. The myocilin response to dexamethasone treatment has been reported as specific for trabecular meshwork cells [18], [19]. Cells were used from passages 4 to 12. All experiments were performed at least three times with similar results.

### Reagents

Antibodies raised against the following proteins were used: MMP-2, MT-1-MMP, TIMP-1, GAPDH (Millipore, Schwalbach, Germany), PAI-1 (R&D Systems, Wiesbaden, Germany), Nck-1 (abcam, Cambridge, UK), Grb2 (Santa Cruz Biotechnology, Heidelberg, Germany) Alexa-568-conjugated goat anti-rabbit (Life Technologies, Darmstadt, Germany), HRP-conjugated secondary antibodies (Jackson/Dianova, Hamburg, Germany). Human recombinant TGF-β2 (Peprotech, Hamburg, Germany) was used at 2 ng/ml. The ROCK inhibitor H1152 is a derivative of HA-1077 and was used for its improved binding properties and high selectivity [20] at 5 or 10 µM as indicated, to ensure a sustained effect in long-term incubations (Calbiochem/Merck, Frankfurt, Germany).

### ECM Degradation Assay

Localized gelatinolytic activity was assessed as described by Ayala et al. [21] with minor modifications. In brief, glass coverslips were coated with Oregon-green tagged gelatine (Molecular Probes) for 1 h at 37°C, washed with PBS, crosslinked with 0.5% glutardialdehyde for 3 min on ice, washed with PBS, treated with 5 mg/ml NaBH4 for 3 min at room temperature, washed in PBS, incubated in 70% ethanol for 10 min, washed and incubated in cell culture medium at 37°C.

HTM cells were plated in the presence of a broad-range MMP-inhibitor (MMP inhibitor II, Calbiochem, 1 µM f.c.) and allowed to attach over night. To synchronize the start of gelatinolysis, the MMP inhibtor was removed by washing with regular medium, and the cells were allowed to digest the substratum for 24 hours. Next, the cells were fixed in 2% PFA, permeabilized using 0.2% Triton X100 and stained for actin, cortactin or fibronectin using phalloidin-TRITC, anti cortactin (Santa Cruz biotechnologies) or anti fibronectin (Sigma) and an Alexa-568-tagged secondary antibody (Molecular Probes). To quantify areas of degradation, at least 6 random fields containing at least 100 cells were assessed. Image J software [22] was used to measure digested area size and cell-covered area.

### Western Blot

Cells were lysed on ice in RIPA lysis buffer (20 mM TRIS, 150 mM NaCl, 0.1 mM EDTA, 1% Triton X-100, 1% Deoxycholate, 0.1% SDS) containing phosphatase and protease inhibitors (Phosphatase Inhibitor Cocktail III, Calbiochem/Merck, Bad Soden, Germany; Complete Protease Inhibitor, Roche, Mannheim, Germany), subjected to SDS polyacrylamide gel electrophoresis, transferred onto a PVDF membrane (Amersham, Braunschweig, Germany) and probed with antibodies against MMP-2, MT-1-MMP, TIMP-2 and GAPDH.

### Zymography

Cells were plated at confluent densities, allowed to adjust for 7d, starved for 3d in 0.5% FCS in the presence or absence of TGF-β2 (2 ng/ml) and subsequently kept in serum-free DMEM (±TGF-β2, H1152 as indicated) for 15 h. Subsequently, conditioned medium was collected, proteins were concentrated using centricon spin columns (Millipore, Schwalbach, Germany) and the cell layer was harvested for Western Blot as above. Conditioned medium proteins (15 µg total protein/lane) and a recombinant human MMP-2 positive control (60 ng protein/lane, Sigma) were separated on a zymography gel containing 12% acrylamide and 0.02% gelatine, treated in renaturing buffer (2.5% TX-100 in water) and developing buffer (Bio-Rad Laboratories, München, Germany). Finally, coomassie staining was used to visualize areas of gelatinolysis.

### RT-qPCR

Cells were plated at confluent densities, allowed to adjust for 7d, treated with TGF-β2 (2 ng/ml) or vehicle for 3 days, rinsed with PBS, and harvested using the RNeasy kit (Qiagen, Hilden, Germany) as recommended by the manufacturer. Two µg of extracted RNA were reverse transcribed (Superscript II, Qiagen) using Oligo-dT primers (Promega). Primer pairs (Table 1) were designed using Primer 3 software [23] (http://frodo.wi.mit.edu/primer3/input.htm). A commercially available kit (SYBR Premix Ex Taq II, Takara Bio Inc., Otsu, Japan) was used for SYBR-green-monitored real-time PCR amplification performed in triplicates on a Step One plus cycler (Applied Biosystems, Foster City, U.S.A.). Enzyme activation (95°C, 20 s) was followed by 40 cycles of denaturation (95°C, 5 s), primer annealing (see table 1, 10 s) and primer extension (60°C, 20 s). mRNA levels were determined as CT threshold levels and normalized with the individual HPRT1 control CT values.

**Table 1:** Primer pairs.

transcript	primer A	
	primer B	annealing temp.
Collagen-1A1	GAGAGCATGAC-CGATGGATT	
	CCTTCTTGAGGTTGCCAGTC	54
Collagen-4A1	GGTATTCCAGGATGCAATGG	
	TCTCACCTGGATCACCCTTC	54
Collagen-6A2	AAGGAGAACCTGGGAGGAAA	
	GGTCCTGGGACTCCTCTTG	60
Fibronectin-1	AATCCAAGCGGAGAGAGTCA	
	CATCCTCAGGGCTCGAGTAG	54
HPRT	GACCAGTCAACAGGGGACAT	
	ACACTTCGTGGGGTCCTTTT	54

### Statistics

Statistical analyses were performed as Dunnett contrast post hoc ANOVA using R software.

## Results

### Human Trabecular Meshwork Cells Form Podosomes and Invadopodia

**Figure 1:**
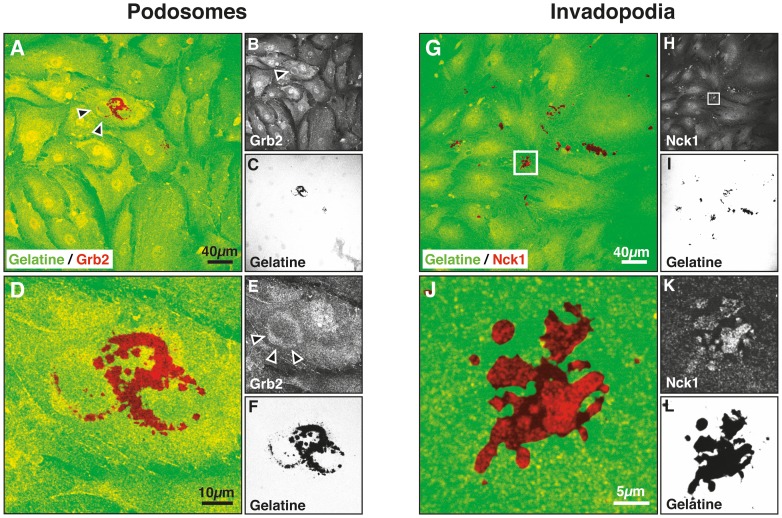
Podosome and invadopodia markers in human trabecular meshwork (HTM) cells. To visualize localized proteolytic activity, HTM cells were plated on fluorescently labelled gelatine, allowed to spread over night in the presence of a MMP inhibitor, washed, incubated in growth medium for 24 h, fixed and stained for the podosome marker Grb2 (A–F) or the invadosome marker Nck1 (G–L). Composite images acquired with a confocal laser scanning microscope reveal a ring-shaped Grb2 signal (red in A, D) which is localized to areolar digestion zones (green in A, D). Furthermore, finger-shaped sites of Nck1 staining coincide with patchy areas of gelatinolysis (J–L).

The formation of podosome- or invadopodia-like structures (PILS) has been suggested in trabecular meshwork cells based on the colocalization of structural proteins and MMP-2 and -14 [15]. To verify the presence of podosomes or invadopodia in a standardized functional assay, we studied localized gelatinolytic activity in MMP-inhibitor washout experiments. Human trabecular meshwork cells were plated on fluorescently labeled gelatine and allowed to spread for 8 h in the presence of an MMP-inhibitor. Subsequently, the cells were washed with cell culture medium to remove the MMP-inhibitor and allow for synchronized onset of gelatinolysis in a confluent cell layer. After 16 h, the cells were fixed and stained for the recently characterized invadosome marker proteins Grb2 or Nck1 (fig. 1). Nck1 and Grb2 differentialy localize to invadosomes or podosomes where they activate WASp proteins to induce Arp2/3-mediated actin nucleation [24]. HTM cells spontaneously formed characteristic ring-shaped structures, which stained for the podosomal marker Grb2 and colocalized with rings of gelatinolytic activity (fig. 1 A–F). Since podosomes are transient dynamic structures with a reported half-life of 2–12 min [25] not all gelatinolytic rings colocalize to a corresponding Grb2 signal at the time of fixation. Spontaneous formation of invadopodia as characterized by Nck1 localization to confluent spots of gelatinolytic activity was also detected in HTM cells (fig. 1 G–L).

### TGF-β2 Enhances Invadosome Formation and MMP-2 Activity in Human Trabecular Meshwork Cells

**Figure 2:**
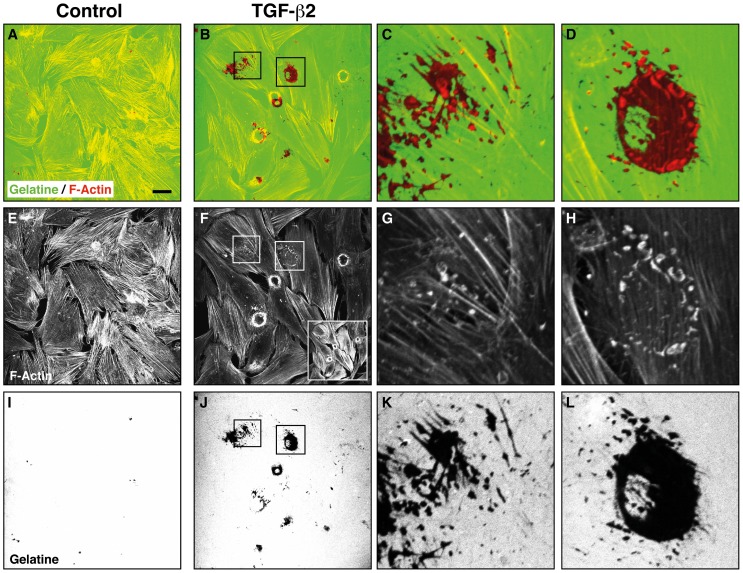
Effect of TGF-β2 on invadosomal proteolysis in HTM cells. The cells were treated with vehicle or TGF-β2 for 3d before plating in the ECM degradation assay. Confocal micrographs depict fluorescently labelled gelatine (green in A–D, grayscale in I–L) and F-actin (red in A–D, grayscale in E–H). TGF–β2 increased F-actin staining intensity (insert in F) and gain was reduced (in F–H) for image acquisition to avoid signal saturation. Scale bar represents 40 µm.

**Figure 3:**
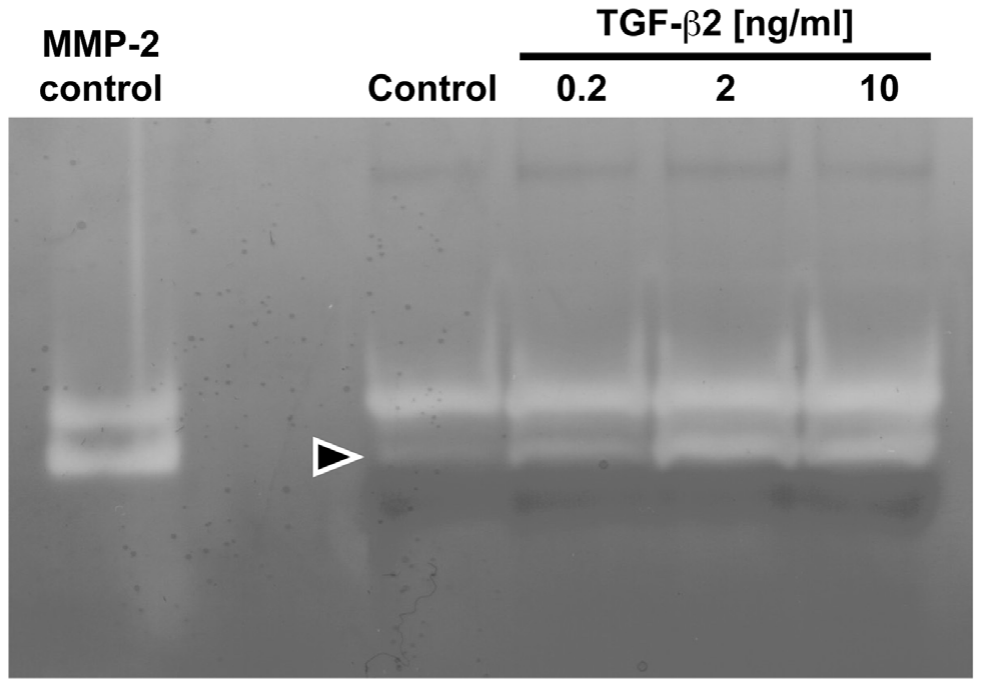
Effect of TGF-β2 on MMP-2 activity in HTM cells. Cells were treated with vehicle or TGF-β2 for 3d, serum-starved for 15 h, conditioned starvation medium was collected, proteins were concentrated using spin-columns and subjected to zymography. MMP-2 renders two bands, a lower one for the cleaved active enzyme (arrowhead) and a higher one for the larger uncleaved protein, as indicated by the positive control (MMP-2 control).

To study the effect of TGF-β on localized ECM degradation, HTM cells were pretreated with TGF-β2 or vehicle for 3d and plated on fluorescently labeled gelatine as above. TGF-β-treated cells exhibited a marked increase in localized gelatinolytic activtity (fig. 2). Ring-shaped podosomal as well as confluent invadopodial digestion zones were detected (fig. 2B, F, J). F-actin stains revealed actin-rings indicative of podosomes which partially colocalized to ring-shaped digestion zones (fig. 2 B, F, J). Close-up views reveal the distinct localization of F-actin clusters (fig. 2 G, H, red in C, D) to sites of ECM degradation (fig. 2 unstained areas in K, L, no green in C, D). We used zymography to further investigate a possible influence of TGF-β on MMP-mediated gelatinolysis in HTM cells. These experiments revealed that TGF-β induced a dose-dependent increase in MMP-2 activity (fig. 3).

### ROCK-Inhibition Blocks TGF-β2-Induced Invadosomes

**Figure 4:**
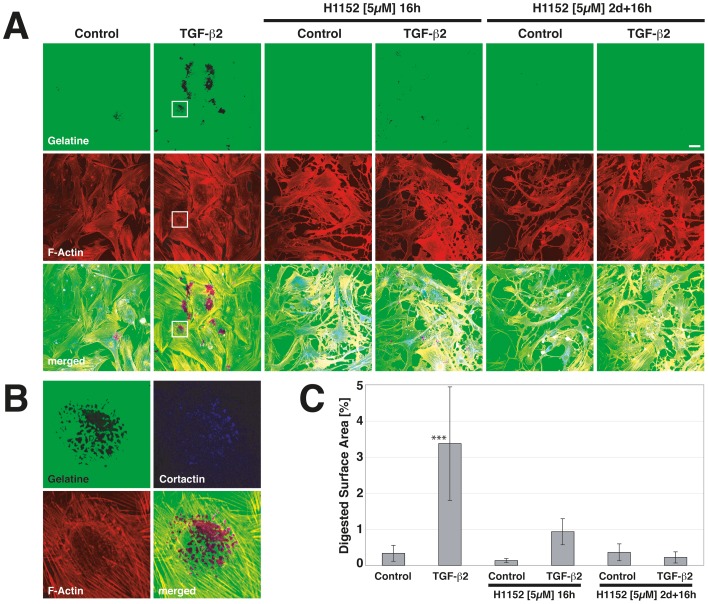
Effect of a ROCK inhibitor on TGF-β-induced invadosomes in HTM cells. (A) ECM degradation assay performed in the presence or absence of TGF-β2 or H1152 as indicated. (B) Close-up of the area marked in (A), depicting colocalization of cortactin and F-actin to sites of ECM degradation. (C) Quantitation of digested surface area in (A). Scale bar represents 40 µm (A), *** indicate p>0.001 in (C).

ROCK inhibitors have been suggested as a novel approach to lower intraocular pressure by decreasing aqueous humor outflow resistance in the trabecular meshwork [7], [26] and recent phase-II clinical trials indicate efficacy [27]. Filamentous actin is a core component of podosomes and invadopodia and ROCK-inhibition induces global rearrangement of the actin cytoskeleton with a loss of actin stress fibers. To investigate a possible effect of ROCK inhibition on invadosome formation, human trabecular meshwork cells were pretreated with vehicle or TGF-β2 for 3d in the presence or absence of a ROCK inhibitor. We used the ROCK inhibitor H-1152 which was reported to have improved binding properties and specificity when compared to the commonly used compound Y-27632 or its precurser molecule HA-1077 [20]. Cells were plated on fluorescently tagged gelatine as above. Invadosome gelatinolysis was allowed for 16 h in the presence or absence of H1152 (fig. 4A, C). TGF-β2 led to a marked increase in invadosomal gelatinolysis (fig. 4, p<0.001 in C) as characterized by the colocalization of cortactin and F-actin to sites of ECM degradation (fig. 4B). In the presence of H1152, TGF-β2-induced gelatinolysis was strongly diminished but still above control levels (difference to control n.s.). Invadosome formation was blocked when H1152 was present during the period of TGF-β pretreatment.

### ROCK-Inhibition Blocks TGF-β2-Induced MMP-2 Activation

**Figure 5:**
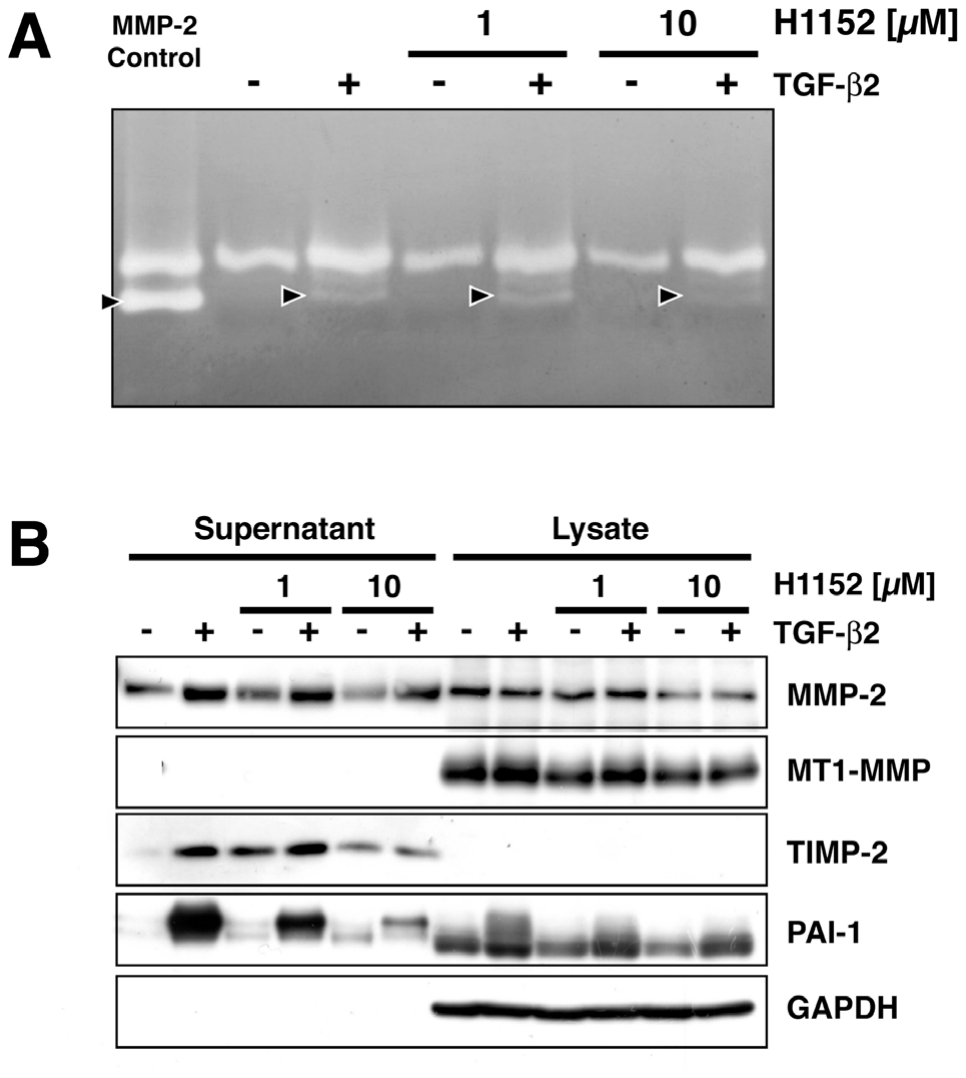
Effect of a ROCK inhibitor on TGF-β-induced MMP-2 activity and protein expression. (A) Zymogram of conditioned media derived from HTM cells treated with vehicle or TGF-β in the presence or absence of H1152. First lane to the left depicts recombinant MMP-2 positive control. (B) Western Blot of conditioned media and cell lysate proteins.

We used zymography and Western Blot to further elucidate a possible effect of ROCK inhibitors on TGF-β-induced changes in the extracellular proteolytic system. TGF-β2-induced MMP-2 expression and activity were reduced by concomitant treatment with H1152 (fig. 5A). Western blot analysis of cell culture supernatants and cell lysates obtained in parallel revealed that TGF-β2 enhanced the expression of MMP-2, MT1-MMP, TIMP-2 and PAI-1 proteins (fig. 5B). This effect of TGF-β2 was dose-dependently reduced by a ROCK inhibitor.

### Increased Invadosomal Activity Coincides with Enhanced ECM Expression

**Figure 6:**
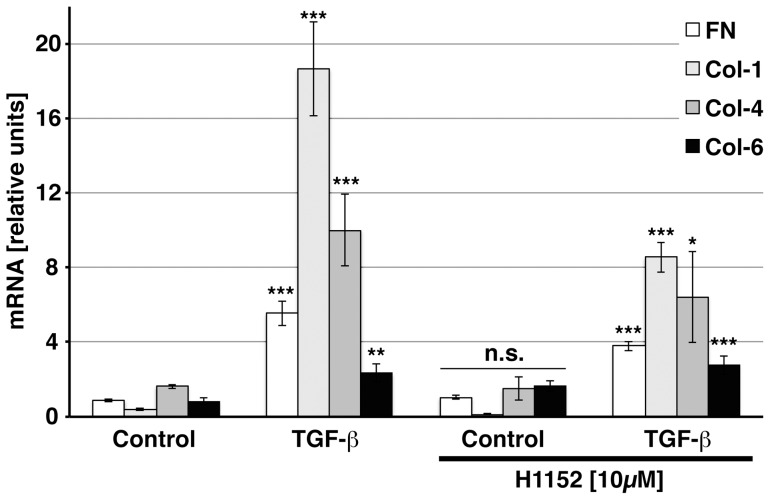
Extracellular matrix protein transcription. HTM cells were treated with vehicle or TGF-β2 in the presence or absence of H1152 for 3d. HPRT-normalized relative transcript levels for fibronectin (FN), collagen-1, -4 and -6 as detected by qPCR. Triplicate means ± SEM. Asterisks indicate significance of difference from controls ***p<0.001, ** p<0.01, * p<0.05; n.s. p≥0.05).

**Figure 7:**
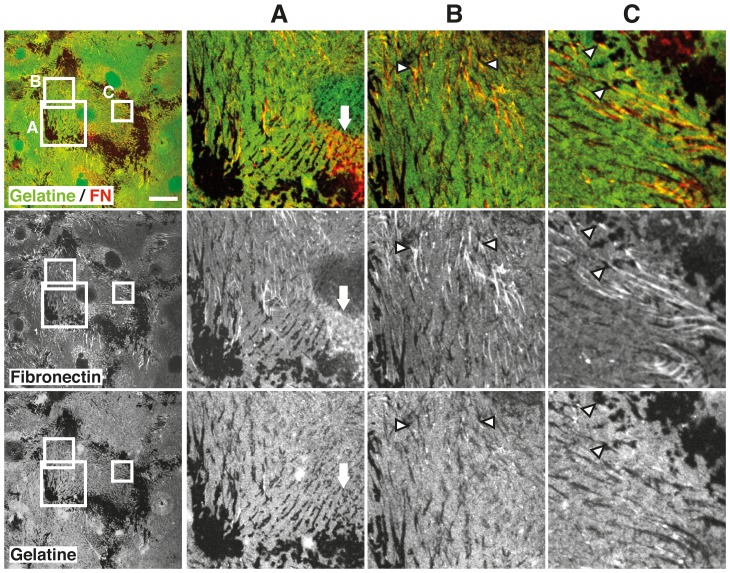
Localization of fibronectin in invadosomal degradation zones. HTM cells were pretreated with TGF-β for 3d, plated in the gelatine degradation assay and stained for fibronectin after 24 h. Confocal micrographs depict localization of gelatine and fibronectin. Close-up views of distinct areas (A, B, C) depict reticular fibronectin in a region of invadsomal gelatinolysis (arrow) and fibrillar strands of fibronectin which appear to originate at the edge of digestion zones (arrowheads). Scale bar represents 40 µm.

To assess ECM deposition in light of increased MMP-activity observed by zymography and fluorescence microscopy, we studied ECM transcription by qPCR. TGF-β2 induced robust increases in transcription of fibronectin and collagens-1, -4 and -6. ROCK inhibition attenuated the effect of TGF-β on fibronectin and collagens -1 and -4, but enhanced baseline and TGF-β-induced expression of collagen-6 transcripts (fig. 6). To further explore possible ECM remodelling processes, we studied fibronectin localization in TGF-β-pretreated cells in the ECM degradation assay. Fibrillar deposits of fibronectin were noted in areas of subtle invadosomal ECM degradation and some of the fibrils appeared to originate at the edges of degradation zones (fig. 7 B, C, arrowheads). In perinuclear areas, a reticular fibronectin signal was present in invadosomal digestion areas (fig. 7 A, arrow).

## Discussion

Primary open angle glaucoma is associated with aberrant ECM deposition in the trabecular meshwork and cell-matrix interactions appear as an essential component of ocular outflow regulation [28], [29]. The molecular mechanisms of trabecular meshwork cell-matrix interactions and their possible disruption in glaucoma are not fully understood. Studies of the distribution of MMPs and cytoskeletal proteins in trabecular meshwork cells in vitro revealed distinct colocalization zones which were termed “podosome- or invadopodia-like structures” (PILS) [15]. Invadosomes are characterized by localized ECM proteolysis [8] and recent data indicate that podosomes and invadopodia can be distinguished based on the presence of distinct proteins involved in actin cytoskeletal modulation [24]. Therefore, we performed ECM degradation assays to clarify this issue. Our data indicate that human trabecular meshwork cells form true podosomes and invadopodia in vitro as characterized by the localization of Grb-2 or Nck-1 to sites of ECM proteolysis (fig. 1).

It has been shown that TGF-β induces podosomal ECM degradation in arterial endothelial cells in vitro [30] as well as in native vascular endothelium [13]. Since intraocular levels of TGF-β2 are increased in primary open-angle glaucoma, we were compelled to assess a possible effect of TGF-β2 on invadosome formation in human trabecular meshwork cells. Cells pretreated with TGF-β2 for 3d showed a marked increase in invadosomal gelatinolysis as compared to vehicle-treated controls (fig. 2), which is in line with observations in other cell types [13], [30], [31]. To further explore this issue, we studied the influence of TGF-β2 on MMP activation in HTM cells by zymography. In our system, TGF-β2 elicited a dose-dependent increase in MMP-2 activation (fig. 3). This is in contrast to an earlier study, which reported TGF-β-induced expression of MMP-2 and PAI-1, but no active MMP-2 was detected either at baseline or following TGF-β stimulation [6]. Only when PAI-1 was blocked by an antibody, active MMP-2 was detected in TGF-β-treated cells. Based on this indirect evidence, a TGF-β -induced, PAI-1-mediated inhibition of MMP activity in HTM cells was suggested and is thought to have a pathophysiologic role in glaucomatous ECM deposition in the trabecular meshwork. We used ultrafiltration columns to improve yield in supernatant protein sample preparation and our detection of active MMP-2 in control conditions suggests a low detection threshold that may have allowed us to observe direct effects on active MMP-2 which may otherwise be missed. The observation of TGF-β-induced MMP-activation in other cell types [30] supports our data and also suggests the concept of localized ECM degradation as integral component of a TGF-β -induced remodelling process.

TGF-β activates RhoA signaling and Rho-kinase (ROCK) inhibitors were shown to block TGF-β-induced transdifferentiation processes [32], [33]. Furthermore, ROCK inhibitors have been reported to decrease ocular outflow resistance and intraocular pressure [7], [26]. In light of these observations, we studied the influence of a ROCK inhibitor on TGF-β-induced changes in proteolysis and invadosomal activity in HTM cells. Inhibition of ROCK induced cytoskeletal changes and a loss of polarity in lamellipodia formation as has been reported earlier [34]. Localized gelatinolysis was markedly reduced in the presence of H1152 and blocked when the substance was applied throughout the period of TGF-β pretreatment (fig. 4). Furthermore, inhibition of ROCK diminished TGF-β-induced expression of PAI-1 and TIMP-2 proteins as well as MMP-2 activity as detected by Western Blot and zymography (fig. 5). The role of ROCK in invadosome formation is not entirely clear. ROCK is activated by Rho-A, -B and -C. It has been demonstrated that Rho-A is required for invadosome formation [35]. Recently, a dramatic decrease in invadopodial matrix degradation has been reported following siRNA-mediated Rho-A depletion in a rat breast cancer cell line [36]. Furthermore, Rho-C was found to regulate cofilin activity to allow for a spatially coordinated focused protrusion of invadopodia [36]. Rho-A and Cdc42 were also reported to control invadopodial delivery of MT1-MMP by modulating interactions of IQGAP and the exocyst complex [37]. ROCK-2 has been localized to invadopodia in intestinal cancer cells and siRNA-mediated knock-down of ROCK reduced MMP-2 activity and invasion [38]. These data are in line with our observations and collectively illustrate an essential role of Rho-GTPases and downstream mediators in invadosomes.

To gain further insight into effects on several proteins involved in proteolysis, we performed zymography and western blot in parallel (fig. 5). TGF-β2 increased levels of MMP-2, TIMP-2, PAI-1 and active MMP-2 in conditioned media as determined by Western Blot and zymography, respectively. Cell lysates revealed a TGF-β-induced increase in PAI-1 and a slight increase in MT1-MMP. It thus appears that proteolyis enhancement by TGF-β is accompanied by expression of counteracting proteins in a negative feedback system. At 10 µM, the ROCK inhibitor H1152 decreased active MMP-2 and TGF-β-induced expression of TIMP-2 and PAI-1.

TGF-β is known to promote extracellular matrix deposition in trabecular meshwork cells [5], [39], [40]. As we had detected increased proteolytic activity following TGF-β treatment in our system, we were compelled to explore the effect of TGF-β on ECM transcription in these conditions (fig. 6). Fibronectin and collagens-1 and -4 were induced by TGF-β and inhibition of ROCK had little effect on baseline expression, but diminished the effects of TGF-β. Collagen-6 transcription was slightly enhanced in the presence of H1152 (fig. 6), which is reminiscent of changes observed in HTM cells plated on soft substrata [41]. Both conditions are associated with decreased adhesion signaling which has an effect on gene transcription. To further assess a possible coincidence of ECM digestion and deposition, we studied fibronectin localization in the context of the ECM degradation assay. This approach has limitations since the optimal time points for the demonstration of gelatine digestion (several hours) and fibronectin deposition (days) differ in this in vitro system and some diffuse deposition of serum-derived fibronectin may add background signal. However, our data clearly indicate the presence of fibrillar fibronectin structures in areas of ECM degradation (fig. 7) and suggest a spatial relationship of degradation site edges and fibronectin fibrils.

In summary, our data indicate that TGF-β2 induces bona fide invadosomes in human trabecular meshwork cells with increased localized proteolysis and a concomitant enhancement of ECM protein transcription. Inhibition of ROCK counteracts the TGF-β-induced effects. These findings allow to envision TGF-β-induced change as an active remodelling process with localized ECM degradation and protein deposition advancing in a coordinated fashion, rather than a mere buildup of undegraded ECM due to a lack of protease activity. Invadosomes may thus have a decisive role in trabecular meshwork maintenance and in changes observed in primary open-angle glaucoma.
